# Epidemiologic Features, Survival, and Prognostic Factors Among Patients With Different Histologic Variants of Glioblastoma: Analysis of a Nationwide Database

**DOI:** 10.3389/fneur.2021.659921

**Published:** 2021-11-24

**Authors:** Li-Tsun Shieh, Chung-Han Ho, How-Ran Guo, Chien-Cheng Huang, Yi-Chia Ho, Sheng-Yow Ho

**Affiliations:** ^1^Department of Radiation Oncology, Chi Mei Medical Center, Liouying, Liouying, Tainan, Taiwan; ^2^Department of Medical Research, Chi Mei Medical Center, Tainan, Taiwan; ^3^Department of Hospital and Health Care Administration, Chia Nan University of Pharmacy and Science, Tainan, Taiwan; ^4^Department of Environmental and Occupational Health, College of Medicine, National Cheng Kung University, Tainan, Taiwan; ^5^Department of Occupational and Environmental Medicine, National Cheng Kung University Hospital, Tainan, Taiwan; ^6^Department of Emergency Medicine, Chi Mei Medical Center, Tainan, Taiwan; ^7^Departement of Medical Education, Chi Mei Medical Center, Tainan, Taiwan; ^8^Department of Radiation Oncology, Chi Mei Medical Center, Tainan, Taiwan; ^9^Graduate Institute of Medical Science, Chang Jung Christian University, Tainan, Taiwan

**Keywords:** glioblastoma, gliosarcoma, giant cell glioblastoma, histologic variant, epidemiology

## Abstract

**Background:** Glioblastoma (GBM) is the most common primary intracranial malignancy. Previous studies found incidence of GBM varies substantially by age, sex, race and ethnicity, and survival also varies by country, ethnicity, and treatment. Gliosarcoma (GSM) and giant cell glioblastoma (GC-GBM) are different histologic variants of GBM with distinct clinico-pathologic entities. We conducted a study to compare epidemiology, survival, and prognostic factors among the three.

**Methods:** We identified GBM patients diagnosed between 2000 and 2016 using the Taiwan Cancer Registry and followed them using the death registry. Survival was compared among conventional GBM and two histologic variants. The potential confounding factors evaluated in this study included registered year, age, sex, and treatment modality (resection, radiotherapy, and chemotherapy).

**Results:** We enrolled 3,895 patients, including 3,732 (95.8%) with conventional GBM, 102 (2.6%) with GSM, and 61 (1.6%) with GC-GBM. GC-GBM patients had younger mean age at diagnosis (49.5 years) than conventional GBM patients (58.7 years) and GSM patients (61.3 years) (*p* < 0.01). The three groups had similar sex distributions (*p* = 0.29). GC-GBM had a longer median survival [18.5, 95% confidence interval (CI): 15.8–25.3 months] than conventional GBM (12.5, 95%CI: 12.0–13.0 months) and GSM (12.8, 95%CI: 9.2–16.2 months), and the differences in overall survival did not attain statistical significance (*p* = 0.08, log-rank test). In univariate analysis, GC-GBM had better survival than conventional GBM, but the hazard ratio (0.91) did not reach statistical significance (95%CI: 0.69–1.20) in the multivariate analysis. Young ages (≤ 40 years), female sex, resection, radiotherapy, and chemotherapy were factors associated with better survival in overall GBMs. In subtype analyses, these factors remained statistically significant for conventional GBM, as well as radiotherapy for GSM.

**Conclusion:** Our analysis found conventional GBM and its variants shared similar poor survival. Factors with age ≤ 40 years, female sex, resection, radiotherapy, and chemotherapy were associated with better prognosis in conventional GBM patients.

## Introduction

Primary brain tumors account for about 1% of all malignant neoplasms. Glioma is the most common brain tumor, and glioblastoma (GBM) is the most common primary intracranial malignancy in adults, which has a dismal prognosis despite multimodality therapy (1). Previous studies found the incidence of central nervous system tumors in the Western world is higher than that in the Eastern world, and the occurrence is also higher in developed countries compared to less developed countries (2). The incidence of GBM varies substantially by age, sex, race, and ethnicity, and prognosis also vary by country, ethnicity, and treatment (2–5). Ostrom et al. reported that non-Hispanic whites had a higher incidence and lower survival rates compared to individuals of other racial or ethnic groups in the US (3). Chien et al. also found disparities by histologic type and grade of primary malignant brain and central nervous system tumors between the US and Taiwan (2).

Glioblastomas comprise a group of morphologically highly heterogeneous neoplasms, as the original designation “multiforme” implies (6). “Glioblastoma” is synonymous with WHO grade IV astrocytoma, GBM multiforme, or conventional GBM in the previous WHO classification. Variants are subtypes of entities that are sufficiently well-characterized pathologically to take a place in the classification and have potential clinical utility (6, 7). Two histologic variants of GBM are recognized as distinct clinicopathologic entities since the 2000 WHO classification: gliosarcoma (GSM) and giant cell glioblastoma (GC-GBM) (6–8). The variants of GSM and GC-GBM possess distinct histologic identities, which may be relevant for tumor behavior and clinical outcomes. The prognosis of GSM appears to be equal or even worse than that of conventional GBM (9–13). GC-GBN also bears a distinct clinico-pathologic picture, traditionally thought to occur more in younger patients and has better survival (13–16).

According to the literature, GSM accounts for 2–8% of overall GBM patients, while GC-GBM comprises only about 1–5% (11–17). The reported outcome of GC-GBM and GSM are limited in a retrospective hospital database or case series with small patient size. Nonetheless, the differences between GSM and GC-GBM may not be fully evaluated, especially in different countries, in the literature. Therefore, they may not fully reflect the distinct clinical features of GBM variants.

To overcome the limitations associated with low incidence, we used the Taiwan Cancer Registry (TCR) database to study histologic variants of GBM. The aims are to identify epidemiologic features, survival, and prognostic factors of the GBM patients with different histologic variants. Modest, yet clinically meaningfully, differences in the effects of treatment modality may surface with the study of a large series. We also conduct a literature review on the incidence and prognosis of histologic variants of GBM reported on the basis of population-based databases in the world.

## Materials and Methods

### Database Sources

The databases of TCR and Taiwan's death registry from 1996 to 2016 were used in this study. The TCR has been organized and funded by the Ministry of Health and Welfare of Taiwan since 1979. Following the enactment of the Cancer Control Act in 2003, all hospitals are mandated to submit cancer data to TCR. The TCR had to monitor the completeness and audit data quality to assure the accuracy of cancer registration data from hospitals reporting, so lag time for reporting cancer incidence is about 4 years. Additionally, TCR data are subjected to periodic quality control audits. It is also overseen by an advisory board and run by the National Public Health Association, which works to standardize terminology, coding, and procedures for the registry. The TCR covers nearly 99% of the cancer patients in Taiwan and records their related information, including the individual demographics, cancer primary sites, tumor histology, and treatment modality. However, the database did not record the exact date of death before 2000. For research purposes, the Health and Welfare Data Science Center (HWDC) set an integrated database center to help academic usage of these databases with de-identified forms in an anonymous format.

### Definition of Study Subjects

The subjects of this study were selected from patients registered in the TCR between 2000 and 2016, and we identified brain tumor patients with the coding of the International Classification of Diseases for Oncology, third edition (ICD-O-3). The percentage of microscopically confirmed cases of malignant brain and central nervous system tumors was around 90% in TCR. Three histologic types of brain tumor were chosen for comparison: GBM not otherwise specified (ICD-O-3 histology code: 9440/3; noted as conventional GBM in this study), GC-GBM (ICD-O-3 histology code: 9441/3), and GSM (ICD-O-3 histology code: 9442/3). Cases without pathologically confirmed, prior diagnosis of glioma or other brain tumor, and also those that were without required data on the TCR such as the date of registration, diagnosis, or treatment were excluded in our analysis.

### Literature Search Strategy

We conducted a search of literature published between January 1995 and December 2019 in PubMed (National Library of Medicine) using “glioblastoma,” “gliosarcoma,” “giant cell glioblastoma,” or “brain tumor” combined with “population-based” or “epidemiology” as keywords. We included epidemiological studies published in full-text English. Case reports, animal studies, reports of GBMs secondary to other conditions, and reports of surgical or radiological management of GBMs were not included.

### Measurements

The primary outcome in this study was mortality. All study subjects were followed up until death or the end date of the study (December 31, 2016). Mortality was identified using the death registry database. The potential confounding factors evaluated in this study included registered year, age, sex, and treatment modality (resection, radiotherapy, and chemotherapy). The TCR database focuses on new cases and thus does not have information about the definite tumor recurrence. Therefore, we are unable to perform disease-free survival analysis.

### Statistical Analysis

We used Pearson's chi-square tests to evaluate the differences in distributions of categorical variables among patients with conventional GBM, GC-GBM, and GSM and used analyses of variance to evaluate the differences in continuous variables. The survival curves were plotted using the Kaplan-Meier method, and the differences were evaluated using log-rank tests. We used Cox proportional regressions to compared survival among the three groups of patients. Multivariate analysis was performed to identify independent factors associated with survival and adjust for effects of potential confounders. We also conducted stratified analyses by histologic type and paired comparisons using conventional GBM as the reference group. We conducted all statistical analyses using SAS 9.4 (SAS Institute Inc., Cary, NC, USA) and performed statistical tests at a two-tailed significance level of 0.05.

## Results

From the TCR database, we enrolled 3,895 histologically confirmed GBM patients in the final analyses, including 3,732 (95.8%) with conventional GBM, 102 (2.6%) with GSM variant, and 61 (1.6%) with GC-GBM. We found the distribution of these three groups of GBMs was quite similar before and after 2007 (*p* = 0.20) (Table 1). Patients with GC-GBM had a younger mean age at diagnosis than patients with conventional GBM or GSM (49.5 vs. 58.7 or 61.3 years, *p* < 0.01). While 26.2% of GC-GBM patients were in the youngest age group (≤ 40 years), only 12.6% of conventional GBM patients and 6.9% of GSM patients were in this age group (*p* < 0.01). The differences in the distribution of sex among the three groups did not reach statistical significance (*p* = 0.29). Patients with conventional GBM were more likely to receive conservative operation (i.e., incisional biopsy only) compared to GC-GBM or GSM patients (18.8 vs. 6.6 or 5.9%, p < 0.01). Differences in the percentage of patients who received adjuvant radiotherapy among the three groups did not reach statistical significance (*p* = 0.23). However, a higher proportion of GSM patients (72.1%) had undergone adjuvant chemotherapy in comparison with patients with conventional GBM or GC-GBM (48.2 or 57.8%, *p* < 0.01).

**Table 1:** Demographic and clinical characteristics of patients diagnosed with glioblastoma and its histologic variants in 2000–2016, Taiwan.

		**Histologic variants**			
**Characteristics**	**All patients**	**Conventional GBM**	**Giant cell GBM**	**Gliosarcoma**	***p*-value**
**Patients**	3,895 (100.0)	3,732 (95.8)	61 (1.6)	102 (2.6)	
**Period of diagnosis**					0.20
2000–2007	1,647 (42.3)	1,603 (97.0)	17 (0.4)	27 (1.6)	
2008–2016	2,248 (57.7)	2,129 (94.7)	44 (2.0)	75 (3.3)	
**Age at diagnosis** (mean ± SD year)	58.6 ± 16.8	58.7± 16.8	49.5 ± 17.8	61.3 ± 15.5	<0.01
**Age group (year)**					<0.01
≤40	493 (12.7)	470 (12.6)	16 (26.2)	7 (6.9)	
40–70	2,266 (58.2)	2,174 (58.3)	35 (57.4)	57 (55.9)	
≥70	1,136 (29.2)	1,088 (29.2)	10 (16.4)	38 (37.3)	
**Sex**					0.29
Male	2,229 (57.2)	2,136 (54.8)	30 (49.2)	63 (61.8)	
Female	1,666 (42.8)	1,596 (45.2)	31 (50.8)	39 (38.2)	
M/F ratio	1.34	1.34	0.97	1.62	
**Resection** ^**a**^					<0.01
Yes	3,185 (81.8)	3,032 (81.2)	57 (93.4)	96 (94.1)	
No	710 (18.2)	700 (18.8)	4 (6.6)	6 (5.9)	
**Radiotherapy**					0.23
Yes	2,669 (68.5)	2,548 (68.3)	47 (77.1)	74 (72.6)	
No	1,226 (31.5)	1,184 (31.7)	14 (22.9)	28 (27.4)	
**Chemotherapy**					<0.01
Yes	1,902 (48.8)	1,799 (48.2)	44 (72.1)	59 (57.8)	
No	1,993 (51.2)	1,933 (51.8)	17 (17.9)	43 (42.2)	

*CI, confidence interval; GBM, glioblastoma; SD, standard deviation*.

a *Subtotal or gross-total resection, other than biopsy only*.

**Figure 1:**
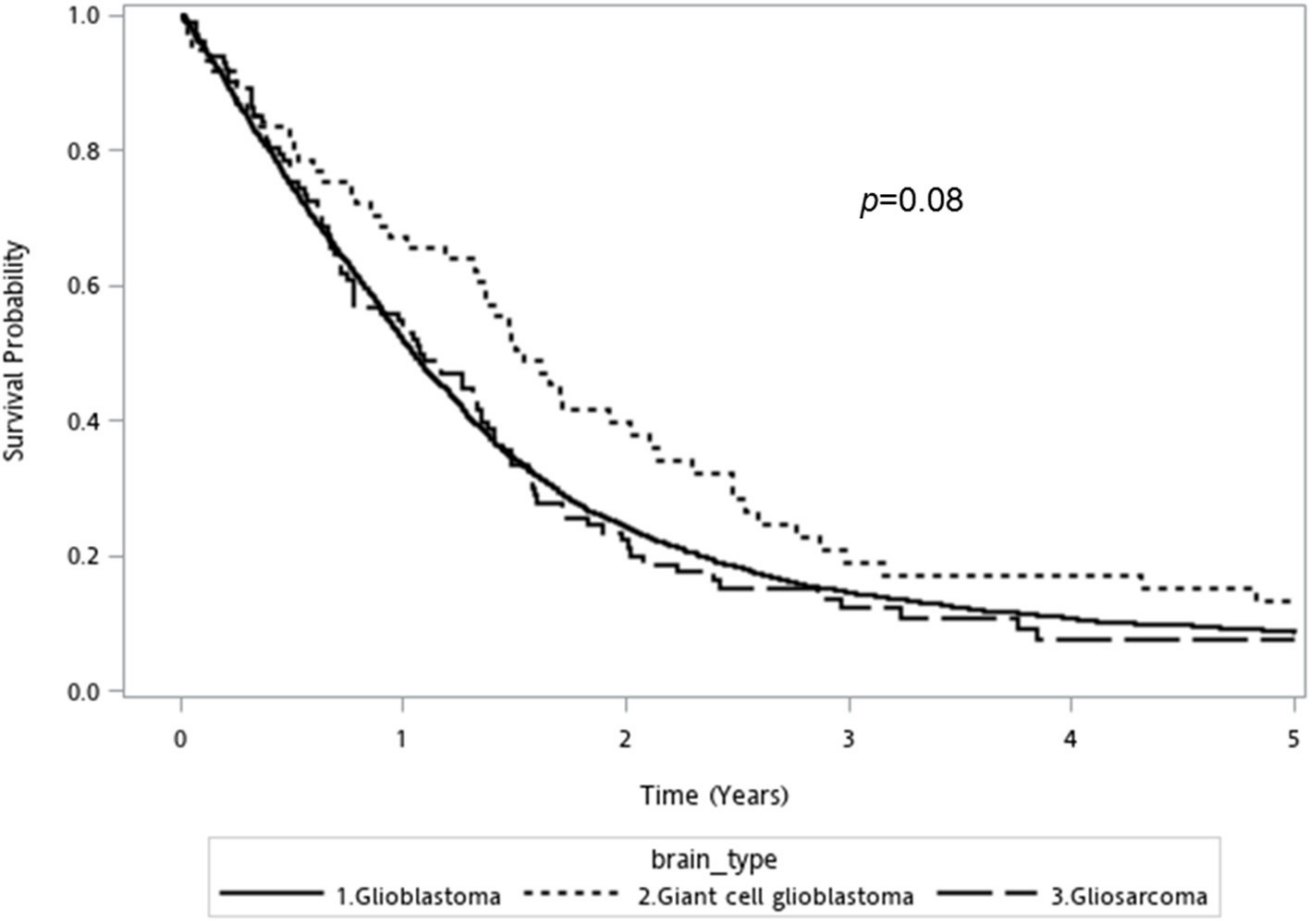
Kaplan-Meier overall survival curve for conventional glioblastoma, giant cell glioblastoma, and gliosarcoma (log rank *p-*value = 0.08).

The prognosis of overall GBM cohort is poor, with an overall median survival of 12.6 [95% confidence interval (CI): 12.1–13.2] months. GC-GBM patients had a median survival of 18.5 (95%CI: 15.8–25.3) months, longer than that of conventional GBM [12.5 (95%CI: 12.0–13.0) months] or GSM [12.8 (95%CI: 9.2–16.2) months]. The 5-year mortality of conventional GBM, GSM, and GC-GBM were 87.9%, 86.3%, and 82%, respectively (*p* = 0.34). The differences in overall survival did not reach statistical significance (*p* = 0.08, log-rank test) (Figure 1).

In univariate analyses, GC-GBM had better survival than conventional GBM with a hazard ratio (HR) of 0.73 (95%CI: 0.55–0.97) (Table 2). Older ages (HR = 1.34, 95%CI: 1.20–1.49 for 40–70 years old and HR = 2.64, 95%CI: 2.35–2.98 for ≥70 years old as compared to ≤ 40 years old) and male sex (HR = 1.16, 95%CI: 1.09–1.24) were also associated with poor survival. Tumor resection (HR = 0.64, 95%CI: 0.59–0.70), adjuvant radiotherapy (HR = 0.51, 95%CI: 0.48–0.55), and chemotherapy (HR = 0.57, 95%CI: 0.53–0.61) were all associated with favorable survival. In multivariate analyses, the 5-year mortality rates of patients with conventional GBM, GC-GBM, and GSM were similar after adjustment for other factors. Nonetheless, younger ages (≤ 40 years) (*p* < 0.01), female sex (*p* < 0.01), resection (*p* < 0.01), adjuvant radiotherapy (*p* < 0.01), and chemotherapy (*p* < 0.01) were shown to be associated with better prognosis of the overall GBM cohort following multivariate analyses (Table 2).

**Table 2:** Univariate and multivariate Cox regression analyses of potential factors associated the survival of overall glioblastoms cohort.

**Variables**	**Univariate**		**Multivariate^**a**^**	
	**HR (95% CI)**	***p*-Value**	**HR (95% CI)**	***p*-Value**
**Variants**				
Conventional GBM	(reference)		(reference)	
Giant cell GBM	0.73 (0.55–0.97)	0.03	0.91 (0.69–1.20)	0.51
Gliosarcoma	1.03 (0.83–1.27)	0.82	1.06 (0.86–1.31)	0.59
**Age at diagnosis (year)**				
≤ 40	(reference)		(reference)	
40–70	1.34 (1.20–1.49)	< 0.01	1.42 (1.27–1.59)	< 0.01
≥70	2.64 (2.35–2.98)	< 0.01	2.56 (2.27–2.88)	< 0.01
**Sex**				
Female	(reference)		(reference)	
Male	1.16 (1.09–1.24)	< 0.01	1.12 (1.05–1.20)	< 0.01
**Resection** ^**b**^				
No	(reference)		(reference)	
Yes	0.64 (0.59–0.70)	< 0.01	0.82 (0.75–0.89)	< 0.01
**Radiation**				
No	(reference)		(reference)	
Yes	0.51 (0.48–0.55)	< 0.01	0.65 (0.59–0.70)	< 0.01
**Chemotherapy**				
No	(reference)		(reference)	
Yes	0.57 (0.53–0.61)	< 0.01	0.72 (0.67–0.78)	< 0.01

*GBM, glioblastoma; HR, hazard ratio; CI, confidence interval*.

a *Adjusted for age, sex, and treatment modality*.

b *Subtotal or gross-total resection, other than biopsy only*.

In stratified analyses by histologic type, older ages (HR = 1.46, 95%CI: 1.30–1.63 for 40–70 years old and HR = 2.58, 95%CI: 2.29–2.92 for ≥70 years old as compared to ≤ 40 years old) and male sex (HR = 1.13, 95%CI: 1.06–1.21) were still independent unfavorable factors for survival in conventional GBMs (Table 3). However, the HRs did not reach statistical significance in GC-GBM or GSM. In fact, a lower HR associated with 40–70 years old was observed for both GC-GBM (HR = 0.69, 95%CI: 0.35–1.38) and GSM (HR = 0.91, 95%CI: 0.38–2.20). Tumor resection was associated with a better survival for conventional GBM (HR = 0.81, 95%CI: 0.74–0.89), but not GC-GBM (HR = 2.49, 95%CI: 0.63–9.93); the HR associated with operation did not reach statistical significance for GSM (HR = 0.74, 95%CI: 0.31–1.81), neither. Radiotherapy was associated with better survival for all three histologic types (HR = 0.65, 95%CI: 0.60–0.71 for conventional GBM and HR = 0.46, 95%CI: 0.28–0.78 GSM), but the HR associated with GC-GBM did not reach statistical significance (HR = 0.51, 95%CI: 0.21–1.21). Chemotherapy was associated with better survival for all three histologic types (HR = 0.73, 95%CI: 0.67–0.79 for conventional GMB), but the associated HR did not reach statistical significance for GC-GBM (HR = 0.51, 95%CI: 0.24–1.07) or GSM (HR = 0.65, 95%CI: 0.40–1.08) (Table 3).

**Table 3:** Univariate and multivariate stratified Cox regression analyses of survival of conventional glioblastoms and histologic variants.

**Variables**	**Conventional glioblastoma**		**Giant cell glioblastoma**		**Gliosarcoma**	
	**Univariate**	**Multivariate**	**Univariate**	**Multivariate**	**Univariate**	**Multivariate**
	**HR (95%CI)**	**HR (95%CI)**	**HR (95%CI)**	**HR (95%CI)**	**HR (95%CI)**	**HR (95%CI)**
**Age at diagnosis (year)**						
≤ 40	(reference)	(reference)	(reference)	(reference)	(reference)	(reference)
40–70	1.37 (1.22–1.53)^*^	1.46 (1.30–1.63)^*^	0.65 (0.34–1.25)	0.69 (0.35–1.38)	0.92 (0.39–2.15)	0.91 (0.38–2.20)
≥70	2.68 (2.37–3.02)^*^	2.58 (2.29–2.92)^*^	1.53 (0.68–3.46)	1.29 (0.40–3.34)	2.33 (0.98–5.57)	2.32 (0.95–5.67)
**Sex**						
Female	(reference)	(reference)	(reference)	(reference)	(reference)	(reference)
Male	1.17 (1.10–1.26)^*^	1.13 (1.06–1.21)^*^	1.05 (0.60–1.83)	1.20 (0.65–2.21)	0.75 (0.49–1.15)	0.87 (0.54–1.40)
**Resection** ^**a**^						
No	(reference)	(reference)	(reference)	(reference)	(reference)	(reference)
Yes	0.64 (0.59–0.70)^*^	0.81 (0.74–0.89)^*^	1.12 (0.35–3.59)	2.49 (0.63–9.93)	0.47 (0.20–1.07)	0.74 (0.31–1.81)
**Radiotherapy**						
No	(reference)	(reference)	(reference)	(reference)	(reference)	(reference)
Yes	0.51 (0.48–0.55)^*^	0.65 (0.60–0.71)^*^	0.50 (0.26–0.94)^*^	0.51 (021–1.21)	0.36 (0.22–0.57)^*^	0.46 (0.28–0.78)^*^
**Chemotherapy**						
No	(reference)	(reference)	(reference)	(reference)	(reference)	(reference)
Yes	0.58 (0.54–0.62)^*^	0.73 (0.67–0.79)^*^	0.28 (0.21–0.70)^*^	0.51 (0.24–1.07)	0.51 (0.33–0.78)^*^	(0.40–1.08)

*HR, hazard ratio; CI, confidence interval*.

* *p-value < 0.05*.

a *Subtotal or gross-total resection, other than biopsy only*.

In paired comparisons, after adjusting for potential confounders, we found GC-GBM and GSM variants have similar survival compared to conventional GBM in all strata by age group, sex, and treatment modality (Supplementary Table).

## Discussion

Glioblastoma comprise a group of morphologically highly heterogeneous neoplasms, as the original designation “multiforme” implies. The cellular composition, even within a GBM tumor *per se*, can vary widely, and mixed histologic features are typical. “Glioblastoma” is synonymous with WHO grade IV astrocytoma in the previous WHO classification or GBM multiforme (6–8). The 2016 WHO classification of central nervous tumors first introduced molecular parameters to define GBM tumor entities, preserved two (GC-GBM and GSM) variants under the umbrella of isocitrate dehydronase (IDH)-wild type GBM. The variants GSM and GC-GBM possess distinct histologic identities, whereas it is seemingly a coherent category of GBM variants, at least based on microscopic tumor morphology alone without regard to biological markers. The GSM variant retains morphologic features of the conventional GBM, while the tumor has differentiated into both biphasic glial and sarcomatous components, and the tumor behavior possesses a higher potential of metastasizing to different lobes of the brain or even to extra-cranial sites clinically. The prognosis of GSM appears to be equal or even worse than that of conventional GBM (9–13). The GC-GBM variant has conventional GBM differentiation, and is further characterized to share a predominance of bizarre multinucleated giant cells and lymphocytic infiltration. GC-GBM also manifests distinct clinical pictures, traditionally thought to occur more often in younger patients and has better outcome compared to conventional GBM (13–16).

We aimed to recruit a large GBM cohort to define the epidemiology and survival factors using the population-based databases in Taiwan. Our results suggest that GSM and GC-GBM variants represent approximately 2.6% and 1.6% of all GBM patients in Taiwan, respectively. Surveillance, Epidemiology, and End Results (SEER) in the US showed that GSM and GC-GBM only accounted for 2.2% and 1% of overall GBM (11, 14), and the ratio of GBM variants National Cancer Database and SEER in the US reported 2.2%–2.9% for GSM and 0.8%–1% for GC-GBM (11, 13–17). The GSM variant, like conventional GBM, shows a propensity to affect the elderly, with a median age at diagnosis around 60 years old in Taiwan and the US. Similarly, both GBM and GSM variants demonstrate comparable male predominance. The occurrence of GC-GBM in Taiwan tended to occur in younger patients with a mean age at 49.5 years, in contrast to 51–56 years in the US (11, 13–17). Nonetheless, with regard to the Asian or Chinese population, reported data on GBM variants were limited. From a hospital-based series, 51 GSM patients were identified with slightly male predominance (59%) and younger age (median age 45 years) in 518 GBM patients at a Chinese hospital (18). The report showed a higher incidence of GSM (9.8%) and a younger age compared to our analysis. However, the hospital-based study might not have sufficient power to precisely characterize Asian GBM variants. Our study is population-based, which enhances generalizability relative to *ad-hoc* hospital-based case series.

Glioblastoma GSM is the most deadly primary brain tumor, with a 5-year survival rate of only about 5%–10%, there are no clinical or pathologic stage classifications of GBMs that are generally accepted (5, 6, 19). Conventional GBM and its histologic variants had a similar worse outcome in our study, and the 5-year mortality rates (87.9% for conventional GBM, 86.3% for GSM, and 82.0% for GC-GBM) are in line with the reported literature (9–19). Our database analyses found GC-GBM patients had a higher median survival of 18.5 months, compared to conventional GBM (12.5 months) and GSM (12.8 months). Our population study had slightly lower median OS than that reported by Stupp et al. (12.6 vs. 14.6 months) (20). Differences in eligibility for the clinical trial in the study by Stupp et al. and the inclusion criteria in our population-based study might explain the discrepancy. In univariate analysis, GC-GBM was found to be associated with a 27% lower risk of mortality in comparison with conventional GBM, but the difference was not significant in multivariable analysis. The prognosis of GC-GBM variant and conventional GBM was found to be equally poor in a review of cases series and hospital-based cancer database (6, 7). However, other studies found a slightly better prognosis for the GC-GBM variant in comparison with conventional GBM (11, 15). The US cancer registry study reported median survival of 11–15.5 months in GC-GBM patients, which is better than conventional GBM in the period of 1988–2004, 1998–2011, and 2004–2014 reported from US SEER or National Cancer Database, respectively (13–15). However, an analysis of the US SEER database of years 1985–2014 showed that GC-GBM and conventional GBM shared similar poor prognosis (16). This indicates that a longer study period with a large sample size might reflect the true outcome between the GC-GBM and GBM cohort.

Some previous studies reported that GSM had a similar survival to conventional GBM, or even worse (9, 10, 12, 13). Our survival analysis showed that GSM and conventional GBM had similar poor overall survival (12.8 vs. 12.5 months). The US cancer registry database showed that, the median survival for GSM was 9–10.7 months (11–13, 17). A review article identified 219 cases of GSM from 34 reports before 2010 and found survival ranging from 4 to 11.5 months. This review provided distinct clinical and pathogenetic features of GSM, including increased metastatic dissemination and worse prognosis than conventional GBM (10). Our analysis is also in line with our previous study that showed no difference in survival between conventional GBM and GSM, and two GSM cases progressed to intra- or extra-cranial metastasis (21, 22). A Chinese study reported a similar median overall survival between GSM (13.0 months) and conventional GBM (14.0 months) (18). Despite the dismal outcome of GSM, adjuvant radiotherapy was found to be an independent predictor in the study.

To the best of our knowledge, this is the first population-based study in Asian patients. Therefore, this is a useful study looking into these rare histologic variants of GBM especially for patients of Asian nationality or heritage. We conducted a literature review to identify epidemiologic studies on the clinical features and prognosis of GBM variants in the databases of Medline and PubMed. Table 4 listed various epidemiologic reports in different national population-based studies, including the US SEER and National Cancer database, and three national registries from Australia, France, and England. However, the three studies only reported case numbers of the variants of GBM without detailed demographics and outcomes (23–25). The results reported the incidence with 1.3%–2.7% GSM and 0.7%–1.8% GC-GBM in other population-based studies. Our study does provide authoritative demographic and survival information on GBM variants in an Asian population, considering that a majority of studies are from American and European populations.

**Table 4:** Literature review on the epidemiologic data on histologic variants of glioblastoma from population-based cancer registries.

**Registry [year, reference]**	**Period**	**All GBM patients**	**Histologic variants**						
			**Conventional GBM**		**GC-GBM**		**GSM**		**Survival outcomes**
			***N* (%)**	**Median age (year)**	***N* (%)**	**Median age (year)**	***N* (%)**	**Median age (year)**	**Median survival (month)**
US, SEER, [2009, (11)]	1988–2004	16,388	16,035 (97.8)	62	–	–	353 (2.2)	63	NS 9 (GBM) 8 (GSM)
US, SEER, [2009, (14)]	1988–2004	16,430	16,259 (99.0)	62	171 (1.0)	51	–	–	GC-GBM > GBM 8 (GBM) 11 (GC-GBM)
Australia, [2011, (23)]	2000–2008	2,275	2,197 (96.5)	–	17 (0.7)	–	62 (2.7)	–	–
US, NCDB, [2014, (13)]	1998–2011	69,935	67,509 (96.5)	61	592 (0.8)	56	1,834 (2.6)	61	GC-GBM > (GBM, GSM) 9.8 (GBM) 13.4 (GC-GBM) 8.8 (GSM)
US, NCDB, [2018, (17)]	2004–2013	37,760	36,658 (97.1)	61.7^a^	–	–	1,102 (2.9)	61.1^a^	NS 11.9 (GBM) 10.7 (GSM)
England, [2018, (24)]	1995–2015	37,786	37,046 (98.0)	–	263 (0.7)	–	477 (1.3)	–	–
US, SEER, [2019, (16)]	1985–2014	25,117	24,909 (99.2)	–	208 (0.8)	–	–	–	NS No survival data
US, SEER, [2019, (15)]	2004–2014	79,543	78,860 (99.1)	62	683 (0.9)	57			GC-GBM > GBM 11.7 (GBM) 15.5 (GC-GBM)
France, FBTDB, [2019, (25)]	2008–2015	2,053	1,988 (96.8)	–	36 (1.8)	–	29 (1.4)	–	–
Taiwan, current study	2000–2016	3,895	3,732 (95.8)	58.7^a^	61 (1.6)	49.5^a^	102 (2.6)	61.3^a^	NS 12.5 (GBM) 18.5 (GC-GBM) 12.8 (GSM)

*FBTDB, French brain tumor database; GBM, glioblastoma; GC-GBM, giant cell glioblastoma; GSM, gliosarcoma; NCDB, National Cancer Database; NS, difference not statistically significant; SEER, surveillance epidemiology and end results*.

a *Mean age*.

Currently, all GBM patients underwent tri-modal therapy, including tumor excision, following chemo-radiotherapy as the standard therapy. We found GBM patients survived longer following standard therapy including operation, radiotherapy, and chemotherapy. These findings parallel the literature, which all confirmed more favorable survival through tri-modality therapy (6, 20, 26). However, regarding GSM patients, we only found the radiotherapy was associated with favorable survival. For GC-GBM patients, we were unable to find favorable prognostic predictors, even following operation, radiotherapy, or chemotherapy. Due to the rarity, even TCR case number might not be sufficiently powered to precisely characterize GSM and GC-GBM. To sum up, the clinical implications of the prognosis of GC-GBM or GSM might share similar or different risk factors compared with conventional GBM. So, the best management of the two rare entities (GSM and GC-GBM) should be further investigated in future clinical trials with hints taken from the epidemiologic study.

The 2016 WHO classification of central nervous tumors introduced molecular parameters in addition to histology to define GBM tumor entities (7). Although there is a trend to incorporate molecular markers into the classification of GBM, none of the markers has been introduced to routine clinical practice before 2016. That is why data on such markers from population-based studies are very limited. Taking SEER for example, we found there were no molecular data between 2001 and 2011 (5), which cover most of our study period. Likewise, IDH1 mutation status was not available in the National Cancer Database between 2004 and 2014 (5). We enrolled GBM patients between the period of 2000 and 2016, and the IDH marker was not mandatory for routine pathologic reports during that period and not registered in the TCR database in Taiwan. Due to lack of data, we were unable to evaluate the prognostic significance of these molecular markers in our study. Further, the more recently defined subtype of epithelioid GBM, there were no registered case data in the study.

It is well-known a younger age is significantly associated with good survival in conventional GBM patients in the literature (6, 20, 26–28). A younger age experienced favorable survival in our GBM cohort. In contrast, aging was not a prognostic factor in GSM or GC-GBM. This may indicate GBM variants possibly differ in clinical manifestation, or it may reflect the relevant chance of hidden bias from a small case number; so caution is advised in interpreting these results.

In the US analysis of GBM variants, the sex factor revealed conflicting results, with some studies showing the female sex experienced a favorable survival in GSM and GC-GBM, yet others reported no difference (11–17). Nonetheless, the sex disparity was not identified in GSM or GC-GBM variants in the study. However, the female sex was associated with improved survival of GBM in the literature (29–31).

The TCR database, similar to other national databases, lacks accuracy in documenting pre-existing comorbidities, and detailed cancer management, which could all impact the outcome analysis. Genetic factors also contribute closely to the prognosis of conventional GBM and its variants. Due to the lack of data, we were unable to evaluate the prognostic significance of these molecular markers in our study. Furthermore, even with large number of cases (3,895 in total), the case number in some subgroup were relatively small and thus might not able to provide sufficient statistical power. For example, chemotherapy was associated with better survival for both GC-GBM and GSM, and the HRs (0.51 and 0.65) were even smaller than that for GBM (0.73), but did not reach statistical significance.

## Conclusion

Utilizing a large national cohort and literature review, this paper adds more information on the epidemiology of GBM in both Asian and Western populations. We confirmed the similar incidence of GSM and GC-GBM in Asian and Western population. Our study showed GBM and its variants shared similar worse outcomes. Resection, post-operative radiotherapy, and chemotherapy did improve survival in conventional GBM, but had different effects on histologic variants.

## Data Availability Statement

The datasets presented in this study can be found in online repositories. The name of the repository and accession number can be found below: Open Science Framework (OSF), https://osf.io/, HFN3D (project name: histologic variants of glioblastoma in Taiwan).

## Ethics Statement

The studies involving human participants were reviewed and approved by the Chi Mei Medical Center Institutional Review Board (IRB No. 10710-L05). Written informed consent for participation was not required for this study in accordance with the national legislation and the institutional requirements.

## Author Contributions

S-YH contributed to conception and design of the study. C-HH organized the database. C-HH and H-RG performed the statistical analysis. L-TS and S-YH wrote the first draft of the manuscript. H-RG, C-CH, and Y-CH wrote sections of the manuscript. All authors contributed to manuscript revision, read, and approved the submitted version.

## Funding

This study was supported by a grant from Chi Mei Medical Center (No. CLFH10724).

## Conflict of Interest

The authors declare that the research was conducted in the absence of any commercial or financial relationships that could be construed as a potential conflict of interest.

## Publisher's Note

All claims expressed in this article are solely those of the authors and do not necessarily represent those of their affiliated organizations, or those of the publisher, the editors and the reviewers. Any product that may be evaluated in this article, or claim that may be made by its manufacturer, is not guaranteed or endorsed by the publisher.
