# Supplementary material for: Epidemiologic Features, Survival, and Prognostic Factors Among Patients With Different Histologic Variants of Glioblastoma: Analysis of a Nationwide Database
Source: Front Neurol. 2021 Nov 24;12:659921. doi: 10.3389/fneur.2021.659921 (PMC8651548; doi:10.3389/fneur.2021.659921)
Supplement: Supplementary file 1 [file Table_1.DOCX]

Supplementary Material

# Supplementary Table

# Supplementary Table Hazard ratio of mortality: multivariate Cox regression analyses by patient’s stratum for histologic variants *vs*. conventional glioblastoma

| Variables | Giant cell GBM *vs*. conventional GBM (reference) | | Gliosarcoma *vs*. conventional GBM (reference) | |
| --- | --- | --- | --- | --- |
|  | Adjusted HR (95%CI) | *p* value | Adjusted HR  (95% CI) | *p* value |
| Age at diagnosis |  |  |  |  |
| ≤ 40 | 1.47 (0.86–2.54) | 0.16 | 1.37 (0.61–3.10) | 0.45 |
| 40–70 | 0.75 (0.50–1.10) | 0.14 | 0.91 (0.63–1.22) | 0.52 |
| ≥ 70 | 0.98 (0.53–1.83) | 0.95 | 1.24 (0.90–1.72) | 0.19 |
| Sex |  |  |  |  |
| Male | 0.86 (0.57-1.28) | 0.45 | 0.93 (0.71–1.23) | 0.63 |
| Female | 1.08 (0.73-1.59) | 0.71 | 1.23 (0.88–1.73) | 0.22 |
| Resection^a^ |  |  |  |  |
| Yes | 1.01 (0.76–1.35) | 0.94 | 1.07 (0.85–1.33) | 0.58 |
| No | 0.71 (0.22–2.23) | 0.55 | 1.18 (0.53–2.63) | 0.69 |
| Radiotherapy |  |  |  |  |
| Yes | 0.87 (0.63–1.20) | 0.40 | 1.00 (0.78–1.29) | 0.99 |
| No | 1.39 (0.80–2.42) | 0.24 | 1.14 (0.78–1.67) | 0.51 |
| Chemotherapy |  |  |  |  |
| Yes | 0.82(0.58–1.15) | 0.25 | 0.99(0.74–1.33) | 0.95 |
| No | 1.16(0.72–1.87) | 0.55 | 1.11(0.81–1.51) | 0.53 |

# GBM glioblastoma, H R hazard ratio, CI confidence interval.

# ^a^subtotal or gross-total resection, other than biopsy only

.
